# Estrogen and COVID-19 symptoms: Associations in women from the COVID Symptom Study

**DOI:** 10.1371/journal.pone.0257051

**Published:** 2021-09-10

**Authors:** Ricardo Costeira, Karla A. Lee, Benjamin Murray, Colette Christiansen, Juan Castillo-Fernandez, Mary Ni Lochlainn, Joan Capdevila Pujol, Heather Macfarlane, Louise C. Kenny, Iain Buchan, Jonathan Wolf, Janice Rymer, Sebastien Ourselin, Claire J. Steves, Timothy D. Spector, Louise R. Newson, Jordana T. Bell

**Affiliations:** 1 Department of Twin Research and Genetic Epidemiology, King’s College London, London, United Kingdom; 2 School of Biomedical Engineering & Imaging Sciences, King’s College London, London, United Kingdom; 3 Zoe Global Limited, London, United Kingdom; 4 National Health Service, London, United Kingdom; 5 Department of Women’s and Children’s Health, University of Liverpool, Liverpool, United Kingdom; 6 Department of Public Health, Policy and Systems, University of Liverpool, Liverpool, United Kingdom; 7 Department of Women’s Health, King’s College London, London, United Kingdom; 8 Newson Health Menopause & Wellbeing Centre, Stratford-Upon-Avon, United Kingdom; Imperial College London, UNITED KINGDOM

## Abstract

It has been widely observed that adult men of all ages are at higher risk of developing serious complications from COVID-19 when compared with women. This study aimed to investigate the association of COVID-19 positivity and severity with estrogen exposure in women, in a population based matched cohort study of female users of the COVID Symptom Study application in the UK. Analyses included 152,637 women for menopausal status, 295,689 women for exogenous estrogen intake in the form of the combined oral contraceptive pill (COCP), and 151,193 menopausal women for hormone replacement therapy (HRT). Data were collected using the COVID Symptom Study in May-June 2020. Analyses investigated associations between predicted or tested COVID-19 status and menopausal status, COCP use, and HRT use, adjusting for age, smoking and BMI, with follow-up age sensitivity analysis, and validation in a subset of participants from the TwinsUK cohort. Menopausal women had higher rates of predicted COVID-19 (P = 0.003). COCP-users had lower rates of predicted COVID-19 (P = 8.03E-05), with reduction in hospital attendance (P = 0.023). Menopausal women using HRT or hormonal therapies did not exhibit consistent associations, including increased rates of predicted COVID-19 (P = 2.22E-05) for HRT users alone. The findings support a protective effect of estrogen exposure on COVID-19, based on positive association between predicted COVID-19 with menopausal status, and negative association with COCP use. HRT use was positively associated with COVID-19, but the results should be considered with caution due to lack of data on HRT type, route of administration, duration of treatment, and potential unaccounted for confounders and comorbidities.

## Introduction

As the COVID-19 pandemic progresses, it has been widely observed that adult men of all ages are at higher risk of developing serious complications. A recent review of biological sex and COVID-19 has described the male bias in COVID-19 mortality in 37 of the 38 countries that have provided sex-disaggregated data [1]. Of women who develop COVID-19, being post-menopausal has been independently associated with more severe COVID-19 [2]. Epidemiological data from previous coronavirus outbreaks, including SARS-CoV (Severe Acute Respiratory Syndrome Corona Virus) and MERS-CoV (Middle East Respiratory Syndrome Corona Virus) showed the same pattern: among men, morbidity and fatality rates were markedly higher compared to women [3, 4]. Although pregnant women are more likely to be admitted to intensive care or to receive ventilation than non-pregnant women of reproductive age [5], they generally experience milder COVID-19 symptoms than initially expected [5, 6].

A recent animal model study of SARS-CoV suggests that the age and sex differences in COVID-19 symptom severity may be explained by protective immune modulatory effects of sex hormones, particularly estrogen [7]. Females have been shown to be able to mount a stronger immune response to a variety of viral infections because of more robust humoral and cellular immune responses [8–10]. Anti-müllerian hormone (AMH) and estradiol are markers of high ovarian reserve and have been shown to negatively correlate with severity of COVID-19, independent of age, suggesting that pre-menopausal women are somewhat protected against severe COVID-19 [11].

The potential protective effect of estrogen against COVID-19 requires continued and careful evaluation. Here, we investigate whether exposure to estrogen is linked to a reduction in the rate and severity of predicted COVID-19 among women, based on large-scale self-reported data from the UK.

## Methods

The COVID Symptom Study Smartphone Application (“app”) was developed by Zoe Global Limited with scientific input from researchers and clinicians at King’s College London and Massachusetts General Hospital. Launched in the UK on 24^th^ March 2020, it captures self-reported information related to COVID-19 symptoms. On first use, the app records self-reported location, age, and core health risk factors. At this point height and weight are self-reported, allowing calculation of body mass index (BMI). With continued use, participants provide daily updates on symptoms, information on health care visits, COVID-19 testing results, and whether they are self-quarantining or seeking healthcare, including the level of intervention and related outcomes. Individuals without apparent symptoms are also encouraged to use the app.

On 7 May 2020 we asked all female app-users if they are presently taking any forms of hormonal therapies including hormone replacement therapy (HRT), hormonal contraceptives and testosterone (S1 Fig). We also posed questions relating to menstruation and current pregnancy. Patients who indicated they were still having periods were asked about frequency of menstruation. Those who indicated not having periods were asked their age at menopause. The COVID Symptom Study dataset used for this study was obtained from the period of 7 May–15 June 2020, yielding 40 days of data collection from a total of 1.9M women in the UK. From these, 1.6M women had a BMI between 20-35kg/m^2^ and were included in downstream analyses. During the study period, COVID-19 testing was not widely available and the app did not yet direct participants to obtain a COVID-19 test based on symptoms.

### Ascertainment of exposures, disease outcomes and study covariates

Exposures, outcomes and covariates were ascertained from self-reported app data following quality control with purpose-built scripts (https://github.com/KCL-BMEIS/zoe-data-prep). Exposures used in our analyses included women’s menopausal status, and COCP and HRT use. Primary disease outcomes included COVID-19-related symptoms (S1 Table) and predicted COVID-19 positivity based on symptoms, as described in our recent publication [12]. Briefly, a predictive model incorporating age, sex, and four symptoms (anosmia, persistent cough, severe fatigue and skipped meals) where the strongest predictor was anosmia, was associated with positive COVID-19 tests. In the UK cohort, the predictive model showed 65% sensitivity, 78% specificity, a positive predictive value of 69%, and a negative predictive value of 75% [12]. Hospitalisation and respiratory support, defined as supplementary oxygen (+/- ventilation) were used as surrogate markers for disease severity. Self-reported results from nose/throat swab tests were also used as outcome for a subset of the sample who were tested for COVID-19. Reported age, BMI and smoking status were used as covariates in the associations.

Analyses of menopausal status included women aged 40–60 years, with BMI 20–35 kg/m^2^, excluding women taking any form of hormonal therapy. BMI cut-offs were used to exclude confounding with extreme BMI. For each analysis, sample sizes describe women who passed the inclusion criteria. We first compared menopausal women with no periods after the age of 40 and a last period within 5 years, to pre-menopausal women of the same age with regular periods occurring every 3–6 weeks. Second, analyses of COCP use included post- and pre-menopausal women aged 20–45 years, with BMI 20–35kg/m^2^. We compared women taking COCP as their only form of hormonal therapy, to women of the same age taking no form of hormone therapy. Lastly, analyses of HRT use were carried out in post-menopausal women aged 50–65 years, with BMI 20–35kg/m^2^ and last periods reported at age 45–60. We compared women on HRT alone to women receiving no form of hormonal therapy. Extended hormone therapy analyses also considered women who were on HRT or related hormone therapies, including COCP, progestogen therapy, progestogen containing intrauterine systems, or testosterone (S1 Table). Use of estrogen for gender transitioning was excluded from the analyses.

### Association analyses

Binomial generalized mixed models with a log-odds/logit link function were used to carry out association analyses. The models included COVID-19 symptom or outcome as a function of exposure variables and covariates including age, BMI, and smoking status. Exposures included menopausal status, COCP use, and HRT use. Exposure data and symptoms of the disease were coded as ‘1’ for positive (TRUE/yes/severe/significant) responses and ‘0’ for negative (FALSE/no/mild) responses or blank (NA) statements. Subjects with a predicted COVID-19 probability ≥ 50% were considered COVID-19-positive. App users in hospital or home from hospital were coded as ‘1’ for hospitalisation. Those who reported going to the hospital and requiring supplementary oxygen (+/- ventilation) were coded as ‘1’ for respiratory support. App users could upload COVID-19 test results to the app, and women with at least one positive swab test were considered COVID-19-positive in the *tested* COVID-19-positive analyses. Only a small subset of women was tested for COVID-19, and missing data and failed tests were excluded from analyses. Age and BMI were coded as continuous fixed effects, and only women with BMI 20–35kg/m^2^ were included. Smoking was coded as a categorical fixed effect variable with levels ‘0’, ‘1’ and ‘2’ representing never-smokers, ex-smokers and current smokers. Smoking status was missing in a proportion of participants, therefore all analyses including smoking as a covariate were carried out in sample subset **(see** S1 Table**)**.

### Sensitivity analyses

Age is a key risk factor for COVID-19. Age sensitivity analyses were performed to match the mean and median ages of cases and controls for each of the three exposure variables. Analyses were carried out in subsets of app users within 5-year bins for menopausal status and use of COCP and HRT. In menopause, two further sensitivity analyses were also performed for duration since time of last period. Sensitivity analyses included, first, selecting women who had not had a period for at least 12 months, and second, selecting women who had a last period within three years.

### TwinsUK cohort data analysis

At the time of data collection, a subset of app users included research volunteers from the TwinsUK cohort [13]. Here, we considered 270 TwinsUK female twins (mean age 66), who also had existing whole blood DNA methylation data profiled using the Illumina Infinium HumanMethylation450 BeadChip. From the 270 female twins, a subset of 84 participants had previously collected TwinsUK questionnaire data on menopausal status, and a further subset of 75 had information on age at last period.

Whole blood DNA extraction and DNA methylation profiling in these samples have been previously described [14]. Briefly, DNA methylation levels were determined using methylation beta-values [15], which range between 0 (unmethylated CpG) and 1 (methylated CpG). ENmix [16] was used for quality control and minfi [17] was used to exclude samples with median methylated and unmethylated signals below 10.5. Three epigenetic age calculators were applied, DNAm GrimAge [18], DNAm PhenoAge [19] and the original Horvath methylation age [20]. Epigenetic age acceleration measures were obtained from regressing epigenetic age on chronological age, and also included blood intrinsic epigenetic age acceleration (IEAA) and extrinsic epigenetic age acceleration (EEAA) [21]. IEAA is calculated by regressing the Horvath DNA methylation age and cell blood counts to estimate cell intrinsic methylation ageing independent of differences in blood cell counts. EEAA is calculated based on epigenetic ageing measures developed by Hannum et al. [22], up-weighted by the relative proportion of three age-related blood immune cells (naïve (CD45RA+CCR7+) cytotoxic T cells, exhausted (CD28-CD45RA-) cytotoxic T cells, and plasmablasts). EEAA has been proposed to capture aspects of age-related immuno-senescence [21]. A one-sided t-test was applied to compare each of the five age acceleration measures to symptoms and predicted COVID-19 status, and results are presented at nominal significance.

### Ethics

The App Ethics has been approved by KCL University Institutional Ethics Committee REMAS ID 18210 (equivalent to IRB in the United Kingdom), review reference LRS-19/20-18210 and covers this study of oestrogen in female participants. All subscribers provided suitably informed digital consent through the smartphone application. No minors under age 18 were included in this study. For the TwinsUK components, ethical approval was granted by the National Research Ethics Service London-Westminster, the St Thomas’ Hospital Research Ethics Committee (EC04/015 and 07/H0802/84)—equivalent to IRB in the United Kingdom. All research participants have signed informed consent prior to taking part in any research activities.

## Results

The COVID Symptom Study dataset used for this study was collected between 7 May–15 June 2020 and included 1.6M women in the UK (BMI range 20–35kg/m^2^), with all analyses adjusted for age, BMI and smoking status.

### Menopause

We examined the impact of entering menopause on predicted and tested COVID-19-positivity and related symptoms among 152,637 women aged 40–60 years with BMI 20–35 kg/m^2^. Cases were defined as menopausal women currently reporting no periods after the age of 40 and with a last period within the past 5 years, resulting in altogether 44,268 women (S1 Table). Controls were defined as pre-menopausal women with regular periods occurring every 3–6 weeks, resulting in altogether 108,369 controls (S1 Table). Women taking any form of hormonal therapy were excluded.

Menopausal women had a higher rate of predicted COVID-19 (OR = 1.22, 95% CI 1.07–1.39, *p* = 0.003) and a corresponding range of significant differences in symptoms including hoarse voice, skipped meals, muscle pains, and fever (Table 1). Requirement for hospitalisation and respiratory support was not significant, but also showed a positive direction of association. Although there was no significant association between menopausal status and testing COVID-19-positive, the direction of association is consistent with predicted COVID-19 results (Table 1).

**Table 1:** Association of menopausal status to predicted and tested COVID-19 results, self-reported symptoms and indicators of COVID-19 disease severity (hospitalisation/respiratory support) (n = 152,637).

	Beta	SE	*p*-value	OR	95% CI	
					LL	UL
Self-reported symptoms						
Abdominal pain (**)	0.117	0.041	0.005	1.12	1.04	1.22
Chest pain	0.020	0.041	0.631	1.02	0.94	1.11
Delirium	0.114	0.058	0.051	1.12	1.00	1.26
Diarrhoea (**)	0.135	0.042	0.001	1.15	1.06	1.24
Fever (*)	0.119	0.051	0.020	1.13	1.02	1.25
Headache	0.026	0.027	0.337	1.03	0.97	1.08
Hoarse voice (***)	0.207	0.051	4.73E-05	1.23	1.11	1.36
Loss of smell (anosmia)	0.064	0.056	0.250	1.07	0.96	1.19
Persistent cough	0.054	0.046	0.236	1.06	0.97	1.15
Severe fatigue	0.047	0.064	0.467	1.05	0.92	1.19
Severe or significant shortness of breath	0.007	0.106	0.951	1.01	0.82	1.24
Skipped meals (**)	0.128	0.047	0.007	1.14	1.04	1.25
Sore throat	0.038	0.030	0.214	1.04	0.98	1.10
Unusual muscle pains (***)	0.232	0.043	5.91E-08	1.26	1.16	1.37
Hospitalisation	0.043	0.139	0.758	1.04	0.80	1.37
Respiratory support	0.468	0.539	0.386	1.60	0.55	4.59
Predicted COVID-19 (**)	0.198	0.067	0.003	1.22	1.07	1.39
Tested COVID-19^	0.013	0.148	0.929	1.01	0.76	1.35

*: *p* < 0.05;

** *p* < 0.01;

*** *p* < 0.001.

^: in a subset of COVID-19-tested individuals (n = 14,796).

SE: standard error; OR: odds ratio; 95% CI (LL; UL): upper and lower limits for the 95% confidence interval of the odds ratio.

The mean age of menopausal and pre-menopausal women included in the analysis above was 53.8 and 45.2 years, respectively. Because of this difference, sensitivity analyses were performed for age within 5-year age bins (40–45, 45–50, 50–55 and 55–60 years old). Upon subgroup analysis by age, we observed that predicted COVID-19 results were most driven by the 45–50 age group (OR = 1.35, 95% CI 1.05–1.72, *p* = 0.017), wherein anosmia, as well as fever and persistent cough, and the need for oxygen treatment in hospital were also significant (S2 Table). Analyses excluding smoking showed consistent findings.

Women who reported having no periods for at least 12 months made up 97% of menopausal women in our study. Sensitivity analyses restricting to only women amenorrhoeic for 12 months showed consistent results, with symptoms and predicted COVID-19 retaining significance and direction of association (OR = 1.22, 95% CI 1.07–1.39, *p* = 0.003; S2 Table). A further sensitivity analysis was also carried for last period within 3 years at time of reporting. Sensitivity analysis for last menstrual period within 3 years from questionnaire showed similar significant results for higher rate of predicted COVID-19 in post-menopausal women (OR = 1.17, 95% CI 1.01–1.36, *p* = 0.036).

### Use of combined oral contraceptive pill

We examined the link between COCP use and COVID-19-positivity and related outcomes in 295,689 women aged 18–45 years (BMI 20–35). Both pre- and post-menopausal women were considered for this analysis, where most participants (85%) were pre-menopausal. Cases were defined as females on the COCP as their only form of hormonal therapy, resulting in 64,253 COCP-users (S1 Table). Controls were women of the same age and BMI group taking no form of hormone therapy, resulting in 231,436 controls (S1 Table).

Women using COCP had a lower rate of predicted COVID-19 (OR = 0.87, 95% CI 0.81–0.93, *p* = 8.03E-05) and a corresponding reduced frequency of symptoms, including persistent cough, delirium, anosmia, skipped meals, severe fatigue and pain (*p* < 0.001) (Table 2). Rate of hospitalisation was also significantly lower in COCP users (OR = 0.79, 95% CI 0.64–0.97, *p* = 0.023). The association between COCP use and tested COVID-19 as outcome was not significant, but showed a consistent negative direction of association in the 25–30 and 35–40 age groups (S2 Table). Analyses excluding smoking showed consistent results. Analyses in the subset of 251,786 pre-menopausal women alone, including 52,453 COCP-users and 199,333 controls, showed consistent strong negative associations with predicted COVID-19 (OR = 0.87, 95% CI 0.80–0.95, *p* = 8.38E-04) and a corresponding reduced frequency of a wide range of symptoms (S2 Table).

**Table 2:** Association of COCP use to predicted and tested COVID-19 results, self-reported symptoms, and indicators of COVID-19 disease severity (hospitalisation/respiratory support) (n = 295,689).

	Beta	SE	*p*-value	OR	95% CI	
					LL	UL
Self-reported symptoms						
Abdominal pain (**)	-0.072	0.024	0.002	0.93	0.89	0.97
Chest pain (***)	-0.119	0.025	2.29E-06	0.89	0.85	0.93
Delirium (***)	-0.174	0.033	1.13E-07	0.84	0.79	0.90
Diarrhoea	-0.042	0.025	0.092	0.96	0.91	1.01
Fever (*)	-0.070	0.029	0.016	0.93	0.88	0.99
Headache	-0.016	0.016	0.325	0.98	0.95	1.02
Hoarse voice (***)	-0.119	0.033	3.34E-04	0.89	0.83	0.95
Loss of smell (anosmia) (***)	-0.140	0.036	1.17E-04	0.87	0.81	0.93
Persistent cough (***)	-0.094	0.028	7.03E-04	0.91	0.86	0.96
Severe fatigue (***)	-0.145	0.040	2.57E-04	0.87	0.80	0.94
Severe or significant shortness of breath	-0.106	0.064	0.095	0.90	0.79	1.02
Skipped meals (***)	-0.205	0.027	4.50E-14	0.81	0.77	0.86
Sore throat (***)	-0.078	0.018	1.63E-05	0.93	0.89	0.96
Unusual muscle pains (***)	-0.152	0.028	3.75E-08	0.86	0.81	0.91
Hospitalisation (*)	-0.239	0.105	0.023	0.79	0.64	0.97
Respiratory support	-0.811	0.480	0.091	0.44	0.17	1.14
Predicted COVID-19 (***)	-0.144	0.037	8.03E-05	0.87	0.81	0.93
Tested COVID-19^	0.076	0.086	0.380	1.08	0.91	1.28

*: *p* < 0.05;

** *p* < 0.01;

*** *p* < 0.001.

^: in a subset of Covid-19-tested individuals (n = 26,871).

SE: standard error; OR: odds ratio; 95% CI (LL; UL): upper and lower limits for the 95% confidence interval of the odds ratio.

The mean ages of COCP users and controls were 29.5 and 34.2 years, respectively. Sensitivity analyses were performed for age, in 5-year bins (18–25, 25–30, 30–35, 35–40 and 40–45-year-olds). Sensitivity analyses showed strongest results for the 25–30 year age group, with COCP-users having lower predicted COVID-19-positivity (OR = 0.77, 95% CI 0.67–0.90, *p* = 0.0008) and avoiding hospitalisation (OR = 0.5, 95% CI 0.31–0.80, *p* = 0.004) (S2 Table). Negative association with predicted COVID-19 was also significant in the 40–45-year-olds (OR = 0.77, 95% CI 0.63–0.94, *p* = 0.01). Associations in the 18–25-year-olds yielded only two statistically significant symptom associations, suggesting that this age group contributed the least to the results observed in the main analysis.

### Use of hormone replacement therapy

The association between HRT use and COVID-19 was assessed in 151,193 post-menopausal women aged 50–65 years, with BMI 20–35 and last periods reported at age 45–60. Controls were post-menopausal women matching these criteria who received no form of hormonal therapy, resulting in 133,395 controls (S1 Table). Cases were defined as 17,798 women on HRT only. Extended analyses also considered women on HRT or additional related hormone therapies, excluding use of estrogen in gender transitioning (S1 Table).

HRT use was associated with an increased rate of predicted COVID-19 (OR = 1.32, 95% CI = 1.16–1.49, *p* = 2.22E-05) and frequency of a wide range of symptoms (S3 Table). Analyses excluding smoking as a covariate showed similar results. However, while predicted COVID-19 and reporting of symptoms showed positive associations in HRT users, there was no significantly increased rate of hospitalisation in HRT-users. Notably, both the need for respiratory support and testing positive for COVID-19 showed a negative trend of association with HRT use, although the results were not nominally significant. The results remained consistent in extended analyses considering use of HRT or other related hormone therapies (S3 Table).

The mean ages of HRT users ranged between 56.6–56.8 years, while the mean age of controls was 58.2 years. Sensitivity analyses were performed for age, selecting for subgroups of women aged 50–55, 55–60 and 60–65 years. Sensitivity analyses of age were consistent with the overall predicted COVID-19 and symptoms results and showed negative directions of association for testing COVID-19-positive, most consistent in 55–60-year-olds (S2 Table), which yielded more COVID-19 tests than the 50–55 and 60–65-year-old subgroups (S1 Table).

### App data validation in TwinsUK questionnaires

A subset of app users included 270 female research volunteers from the TwinsUK cohort [13], where 84 had previously reported questionnaire data on menopausal status. For all 84, menopausal status in the app response matched the TwinsUK questionnaire data, where women reported that periods had either stopped or that they did not currently have periods. Furthermore, a subset of 75 female twins had reported information on age of last period in TwinsUK questionnaires. Of these, 64% of twins (48 twins) matched age of last period reported from TwinsUK data within 1 year to age at last period reported in the app, and 87% (65) matched within a 3-year range.

### Menopause, biological aging, and COVID-19 symptoms

Menopause is a marker of ageing and has previously been linked to accelerated epigenetic ageing [23]. To this end, we compared the frequency of COVID-19 symptoms in 270 TwinsUK female twins (S4 Table) with available app data to 5 estimates of epigenetic ageing in whole blood, including the original epigenetic age acceleration, GrimAge acceleration, PhenoAge acceleration, blood cell *intrinsic* epigenetic age acceleration (IEAA), and blood *extrinsic* epigenetic age acceleration (EEAA).

Overall, fatigue and unusual muscle pains showed the most (3 or more) nominally significant associations with epigenetic age acceleration measures; followed by hoarse voice, skipped meals and anosmia where significant differences were observed for two age acceleration measures; and fever where a significant difference was observed with GrimAge Acceleration alone (*p* = 0.01) (S5 Table). However, the results should be interpreted with caution as sample sizes for subgroup analyses are modest, and in cases very small. Similarly, the number of individuals with predicted COVID-19 was extremely small (3 predicted cases), but these individuals as a group had on average accelerated epigenetic age acceleration across all five epigenetic ageing measures (S5 Table).

## Discussion

### Summary

As hypothesised, our results show that being pre-menopausal appears to have a protective effect against COVID-19 in a large community survey of female UK app-users. This was supported by a protective effect amongst pre-menopausal women taking the COCP but was not seen for post-menopausal women taking HRT. However, the HRT results should be considered with caution due to lack of data on HRT type, route of administration and duration of treatment. The majority of women in the UK are currently given oral estrogen, which has more risks compared to transdermal estrogen, and may also affect immunity differently [24]. Transdermal estrogen contains E2, which has more beneficial effects on immunity [25]. Women taking HRT have been shown to have lower future risk of cardiovascular disease, obesity and type 2 diabetes, which are all known to be associated with more severe symptoms and higher mortality from COVID-19 [26].

### Strengths and limitations

This study collected data on hundreds of thousands of women resulting in good power to detect association effects. However, the study also has several important limitations.

Predicted COVID-19 was the main outcome of our study because at the time of data collection COVID-19 testing was not widely available in the UK. The symptom-based predictive COVID-19 model had a sensitivity of 65% in a UK-based cohort from the COVID Symptom Study app, and the strongest predictor was anosmia [27]. The individuals on which the prediction model was trained were highly selected as COVID-19 testing was not performed at random when the app was initially launched, although testing criteria have since been extended. Additionally, sampling using an app will under-represent individuals without smartphones, including older participants, and is likely to under-represent those severely affected by COVID-19. Furthermore, some COVID-19 symptoms may differ in population subgroups such as menopausal women and affect model performance. However, the majority of significant results obtained for predicted COVID-19 in our study showed a similar direction of association in the small subset of self reported tested COVID-19 cases.

Further important limitations include effects of unmeasured confounding and potential systematic differences between individuals prescribed hormone therapy, and different types of hormone therapy. Specifically, there may be levels of confounding between risk factors for COVID-19, such as BMI and smoking, and factors influencing the likelihood of being prescribed hormone therapy. For example, in the COCP analyses smoking, obesity and older age (> 35 years) increase the risk of thrombotic events in women using combined hormonal contraceptives [28], and alternatives may be advised. Therefore, in our study, women > 35 years on COCP may be healthier than others of similar age. There is also potential for selection bias where, for example, experiencing specific problems and symptoms influences the likelihood of hormonal therapy use. Although we consider multiple key covariates such as age, BMI, and smoking, these factors likely have more complex effects on the response variable and potentially on other covariates, than the effects that we capture using main effects linear models.

The majority of data in our study are self-reported, and questions on medication use were non-standard, to ease large-scale app-based reporting. Data on type, route, duration, and dose of hormone therapies, were not collected due to difficulties faced collecting very detailed data using an app-based interface. As such, untangling the effect of differing types of HRT was not possible. Most COCPs contain between 20–35 micrograms of ethinylestradiol along with a progestogen, while HRT estradiol doses are generally lower and more physiological. As such, lower estrogen doses and lack of detailed data may have resulted in the lack of effect seen amongst HRT-users. Furthermore, dietary phytoestrogens may affect pre- and post-menopausal women differently [29], but our study could not explore their effect on women in relation to COVID-19 infection rates.

Although, we validated self-reported menopausal status in the subset of participants from the TwinsUK cohort, our study did not directly measure levels of estrogen. Therefore, we could not confirm that serum estrogen levels were lower in post-menopausal women, or explore mechanisms impacted by hormone changes related to COVID-19. Moreover, the absence of periods does not necessarily equate to a diagnosis of menopause, particularly in women in their early 40s. Other limitations relate to reporting bias within both symptoms and test results, as well as potential for survival bias. To ascertain the consistency of our results, we performed multiple cumulative data extracts over the period 7 May 2020–15 June 2020, and observed that association results were consistent throughout, including specifically for predicted COVID-19 associations.

### Comparison with existing literature

Teasing out the precise drivers of mortality in COVID-19, regardless of sex, is difficult. The innate recognition and response to viruses as well as downstream adaptive immune responses during viral infections are known to differ between females and males [9]. It has been well-illustrated that females generally mount greater inflammatory, antiviral, and humoral immune responses than males during viral infections [30], which contributes to better clearance of viruses, including SARS-CoV [7]. This heightened inflammatory response is advantageous in response to infection and sepsis, but is unfavourable in immune responses against self, leading to more autoimmune disease in women compared to men [31, 32]. Additionally, enhanced immunity in females can also result in greater immuno-pathology and tissue damage at later stages of viral disease, such as during influenza A virus infection [33]. In line with these observations, women are at greater risk for developing long COVID, compared to men [34]. Conversely, maternal physiological adaptations to pregnancy usually predispose pregnant women to a more severe course of many infections, including viral pneumonia, with subsequent higher maternal and fetal morbidity and mortality [35], but observational cohort studies in COVID-19 have reported that pregnant women are less likely to show symptoms of fever, shortness of breath, and muscle pains than non-pregnant women of reproductive age [6]. Estrogen levels increase more than 100-fold in pregnancy [36] providing a potential mechanism for the unique resistance to COVID-19. Milder COVID-19 symptomology could also be linked to the physiological changes observed in women over the gestation period.

With ageing, a general decline in immune function is observed—immune-senescence. Several of these changes are gender specific and affect post-menopausal women. Levels of estrogen, for example, 17β-estradiol (E2), are variable during the menstrual cycle, high during pregnancy and low after menopause in females. Progesterone (P4) levels are also very high in pregnant women, and P4 is essential to establish and maintain gestation by limiting local and systemic pro-inflammatory immune responses [37]. While progesterone is considered immunosuppressive, estrogens in general are considered immune-stimulatory [38]. Estrogens exert their effects partly through binding to ERα or ERβ, which are expressed in various types of immune cells, including lymphocytes, macrophages, and dendritic cells [39]. E2 thus affects many components of innate immunity, including the functional activity of innate immune cells that influence downstream adaptive immune responses [9]. This is dependent on hormone concentration, in addition to density, distribution and receptor type found in immune cells. As a consequence, lower circulating estrogen levels due to ageing leads to a dampened immune response in older women. For example, in post-menopausal women, a second peak in Human papilloma virus (HPV) prevalence has been reported [40, 41]. New HPV infections in older women with no sexual activity are thought to be due to reduced immune responses [42]. HIV-1 infection is also increasing in post-menopausal women [43], where a European study found that women over 45 have a 4-fold increased risk of acquiring HIV compared to women under 45 years of age [44]. A recent cohort study of 68,466 patients with COVID-19 from 17 countries found that the fatality risk for women > 50 years receiving estradiol therapy was reduced by more than 50% compared with non-users (OR = 0.33, hazard ratio (HR) = 0.29) [45]. Recent results based on the Oxford-Royal College of General Practitioners (RCGP) Research and Surveillance Centre (RSC) database of 1,863,478 women show that HRT use was associated with a significantly lower likelihood of all-cause mortality in COVID-19 (unadjusted OR = 0.15, 95% CI 0.06–0.37, adjusted OR = 0.22, 95% CI 0.05–0.94, p = 0.041) [46].

### Implications for research and practice

Menopause is a marker of biological ageing in women that has previously been associated with accelerated epigenetic ageing [21]. The associations between menopausal status and COVID-19 positivity and symptom severity may in part be related to biological ageing, rather than reduction in estrogen specifically, although the COCP use results suggest that this is unlikely. To explore this further we tested the association between COVID-19 symptoms and epigenetic ageing rates in a subset of participants from the TwinsUK cohorts. Our results are consistent with increased frequency of COVID-19 symptoms among subjects with accelerated biological ageing, including measures that capture aspects of immuno-senescence (EEAA). This suggests the need for further investigation into the link between biological ageing and COVID-19. Additionally, future studies into the role of estrogen warrant more detailed information on HRT and the COCP in the context of COVID-19. HRT provision and prescribing represent a significant area-of-need in women’s health.

## Conclusion

Our findings indicate a protective effect of estrogen from symptomatic COVID-19, based on positive association of menopausal status with predicted COVID-19, and negative association of COCP use with predicted COVID-19. HRT use was positively associated with COVID-19 symptoms, but the results should be interpreted with caution due to lack of data on HRT type, route of administration, duration of treatment, and potential comorbidities. Further work focussed on gender with hormone profiling in both pre-clinical and clinical settings, as well as on biological ageing, is needed to uncover novel features of the host immune response to SARS-CoV-2 and ultimately result in more equitable health outcomes.

## Supporting information

S1 Fig Questions posed to female participants with possible answers. (TIF)

S1 Table Sample characteristics for different components of the study. (XLSX)

S2 Table Results from sensitivity analyses. (XLSX)

S3 Table Results from HRT analyses. (XLSX)

S4 Table Descriptives of the 270 female TwinsUK participants in the study. (XLSX)

S5 Table Association results from COVID-19 symptoms and epigenetic age acceleration measures. (XLSX)
