# Supplementary figures and images for: Estrogen and COVID-19 symptoms: Associations in women from the COVID Symptom Study
Source: PLoS One. 2021 Sep 10;16(9):e0257051. doi: 10.1371/journal.pone.0257051 (PMC8432854; doi:10.1371/journal.pone.0257051)

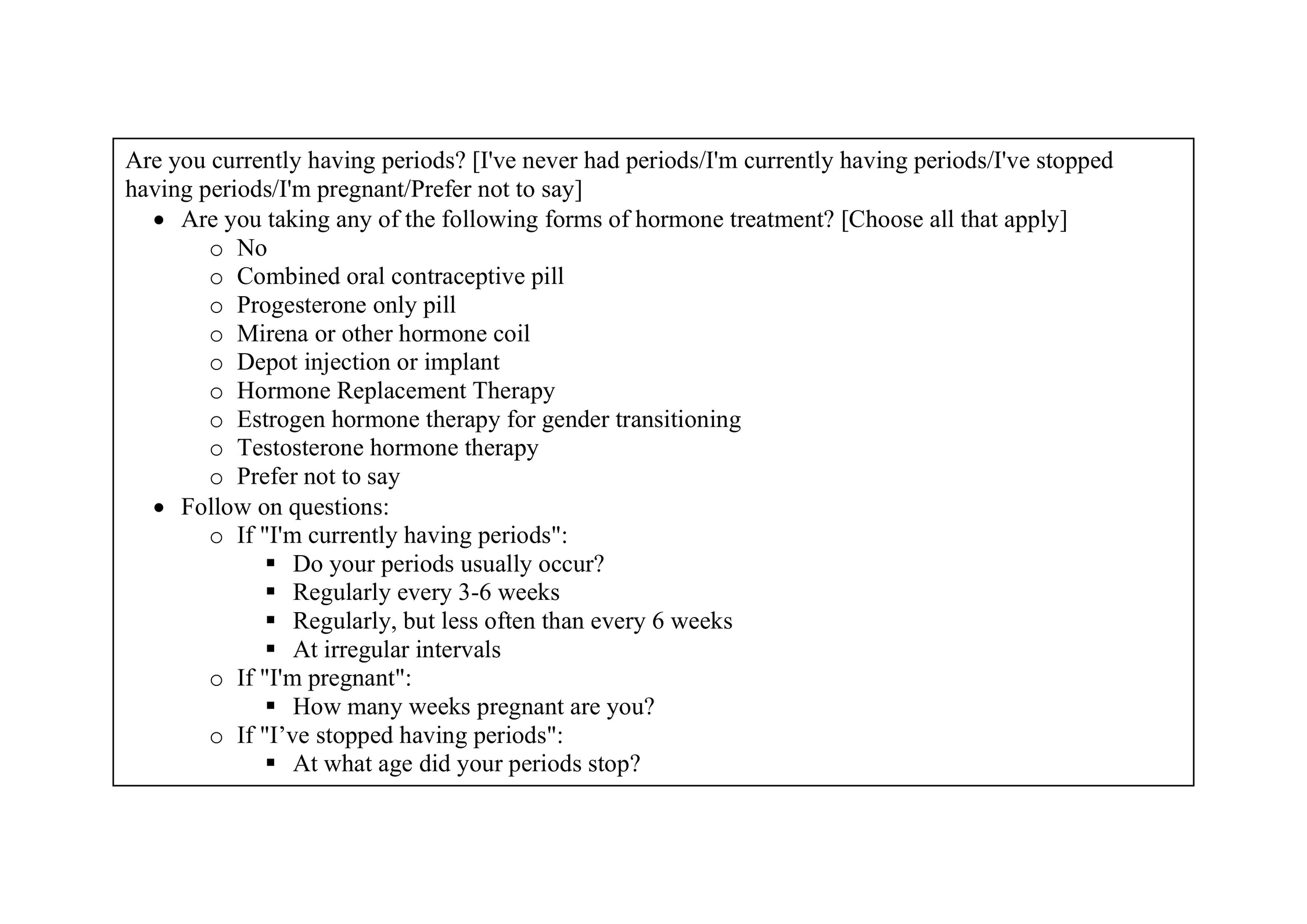

Supplement: S1 Fig — (TIF) [file pone.0257051.s001.tif]
